# Application of the ESRI Geostatistical Analyst for Determining the Adequacy and Sample Size Requirements of Ozone Distribution Models in the Carpathian and Sierra Nevada Mountains

**DOI:** 10.1100/tsw.2001.317

**Published:** 2001-12-07

**Authors:** Witold Fraczek, Andrzej Bytnerowicz, Michael J. Arbaugh

**Affiliations:** Environmental Systems Research Institute, Redlands, California, USA

**Keywords:** air pollution, ozone, geostatistical models, kriging interpolation, prediction error, monitoring network, digital elevation model, GIS, geostatistics

## Abstract

Models of O_3_ distribution in two mountain ranges, the Carpathians in Central Europe and the Sierra Nevada in California were constructed using ArcGIS Geostatistical Analyst extension (ESRI, Redlands, CA) using kriging and cokriging methods. The adequacy of the spatially interpolated ozone (O_3_) concentrations and sample size requirements for ozone passive samplers was also examined. In case of the Carpathian Mountains, only a general surface of O_3_ distribution could be obtained, partially due to a weak correlation between O_3_ concentration and elevation, and partially due to small numbers of unevenly distributed sample sites. In the Sierra Nevada Mountains, the O_3_ monitoring network was much denser and more evenly distributed, and additional climatologic information was available. As a result the estimated surfaces were more precise and reliable than those created for the Carpathians. The final maps of O_3_ concentrations for Sierra Nevada were derived from cokriging algorithm based on two secondary variables — elevation and maximum temperature as well as the determined geographic trend. Evenly distributed and sufficient numbers of sample points are a key factor for model accuracy and reliability.

## INTRODUCTION

The purpose of Geographic Information System (GIS) is to provide a spatial framework to support decisions for the intelligent use of earth's resources and to manage the man-made environment[1]. Biologists, botanists, planners, petroleum engineers, foresters, and corporate executives are increasingly relying on GIS to help them make critical decisions. By putting spatial data in an integrated system where it can be organized, analyzed, and mapped, patterns and relationships that were previously unrecognized may emerge. In the U.S. numerous agencies and companies apply GIS in studies of air pollution. The air pollution continues to be a significant environmental problem in many areas of the country[2].

Geostatistical analysis methods have been available for several decades, but were not integrated into any GIS modeling environments. A newly released software package, Geostatistical Analyst[3] links GIS and geostatistical analysis methods. With Geostatistical Analyst (an extension of the ArcGIS Version 8.1), a continuous surface (a map or a distribution model) can be created from measured sample points. Data collection usually can only be conducted at a limited number of point locations due to logistical and financial limitations, however scientists and managers are increasingly interested in continuous surface estimates. In order to generate such a surface some type of interpolation method must be used to estimate data values for those locations where no samples were taken.

Geostatistical methods, such as kriging apply regionalized variables and describe spatial dependencies between the instances of random variables by using variograms. A variogram is a graphical display of a variance of measurements over the distance between the measurement sites. If there are spatial dependencies the variance between the observations on two points normally increases with increasing distance until at a specific range a maximum value is reached. Kriging is considered to be the most sophisticated geostatistical method as it can potentially provide the most accurate results. Importantly, however, applying kriging is not always straightforward, thus the method must be utilized appropriately and consciously to generate correct outputs.

Although models of $O_3$ distribution have been developed for some areas in the eastern U.S.[4] and Europe[5], no such models based on ground-level monitoring of the pollutant had been developed. Recent development of low-cost passive ozone monitors in recent years[6] have made it practical to develop networks of monitors in remote locations.

In this work an application of the Geostatistical Analyst for development of $O_3$ distribution models will be discussed using two different data sets. In the first data set ozone ($O_3$) concentrations were measured during the 1997-1999 growing seasons in the Carpathian Mountains in Central Europe[7], and in the second data set ozone concentrations were measured during the 1999 growing season in the Sierra Nevada Mountains of California. In both studies concentrations of $O_3$ were measured with passive samplers. In the Carpathian Mountains the monitoring network was relatively sparse, while the network in the Sierra Nevada was much denser.

## METHODOLOGY

### Ozone Monitoring

Passive samplers (Ogawa, Pompano Beach, FL) were used for monitoring ozone concentrations[6]. The samplers were exposed for 2-week long periods during the growing season (May 1 through September 30) and were located in well-exposed forest clearings at about 2–3 m above the ground level. A rate of $NO_3^-$ formation (amount of $NO_3^-$ formed on a filter over time of exposure) served as a measure of $O_3$ concentration. Rates of $NO_3^-$ formation on passive sampler monitors were compared with real-time $O_3$ concentration measurements with the UV absorption Thermo Environmental Model 49 monitors at two sites in the Carpathians and 9 sites in the Sierra Nevada. Empirically derived coefficients from comparison of results from the collocated active and passive monitors were used for calculating $O_3$ concentrations for all passive sampler sites. Ozone concentrations are expressed as ppb (1 ppb $O_3$ at 25°C and 760 mm Hg = 1.96 µg/m$^3$). Precision of the $O_3$ passive samplers was in general less than 5% of the estimate. Four replicate samples were collected for every monitoring period at each location in the Carpathian study and two replicates in the Sierra Nevada study. In the Carpathians the samplers were exposed for 2-week long periods between May 1 and September 30 during the 1997, 1998, and 1999 seasons at 32 monitoring sites. In the Sierra Nevada Mountains, 93 monitoring sites were established and samples were collected from 2-week long exposures between May 1 and September 30, 1999.

In the Sierra Nevada, meteorological data from a network of 63 weather stations, including measurements of maximum daily temperature over the sampling period was used for development of $O_3$ concentration models.

### Geostatistical Analysis

Maps showing spatial distribution of $O_3$ concentration were created with the Geostatistical Analyst extension to ArcGIS 8.1 software produced by Environmental Systems Research Institute (ESRI), Redlands, California[3]. The Geostatistical Analyst uses sample points at different locations in a landscape and interpolates the values measured at these sites into a continuous surface. Using this approach, spatial models of prediction or estimation of $O_3$ concentrations were derived. The Geostatistical Analyst provides a comprehensive set of tools for data exploration and for creating surfaces that can be used to visualize, analyze, and understand the geographic phenomena of interest.

## RESULTS AND DISCUSSION

### Carpathians – The Foundation for Further Research

#### *Study Location and the Data Sources*

General location of the Carpathian Mountains in Europe is shown in Fig. 1. For this study, the delineation of the Carpathian Mountains study area was based on landscape elevation above sea level, geology of the outcrop formations, and land use pattern, especially the lower extent of mountain forests. A final determination of the Carpathians outline was driven by a principle of including all of the major mountain forest stands. As a result, the entire study area was 158,000 $km^2$ and included 107,740 $km^2$ (68.2% of the area) of coniferous, mixed and deciduous forests.

The distribution of 32 monitoring sites in the Carpathian Mountains was uneven (Fig. 2) due to various reasons (mainly available funding). The density of points was much higher in the Western Carpathians than in the Southern Carpathians. However, an effort was made to generate continuous surfaces of $O_3$ concentration for each semi-monthly measurement period as well as for each averaged year for the Carpathian Mountains area. Because the beginning of the monitoring season varied between the sites (partly due to the fact that some locations were not accessible early in a season), the average values of $O_3$ concentrations were calculated for measurements starting on June 15 and ending on September 15. During that time all monitoring sites were fully operational. In order to evaluate an effect of elevation on ozone concentrations, two transects were established along elevational gradients through Lysa Hora in the Morava-Silesian Beskid Mountains near the Czech-Slovak border (Fig. 3). Although no relationship between elevation and $O_3$ concentrations was seen on the NW-SE transect, an increase of $O_3$ concentrations with elevation was clear on the SE-NW transect; for the 1997 and 1998 seasons an increase was logarithmic, and for the 1999 season linear. On the basis of this evidence, we decided that elevation would be used as a covariate for estimation of $O_3$ concentration distribution with the Geostatistical Analyst using the geostatistical method of cokriging.

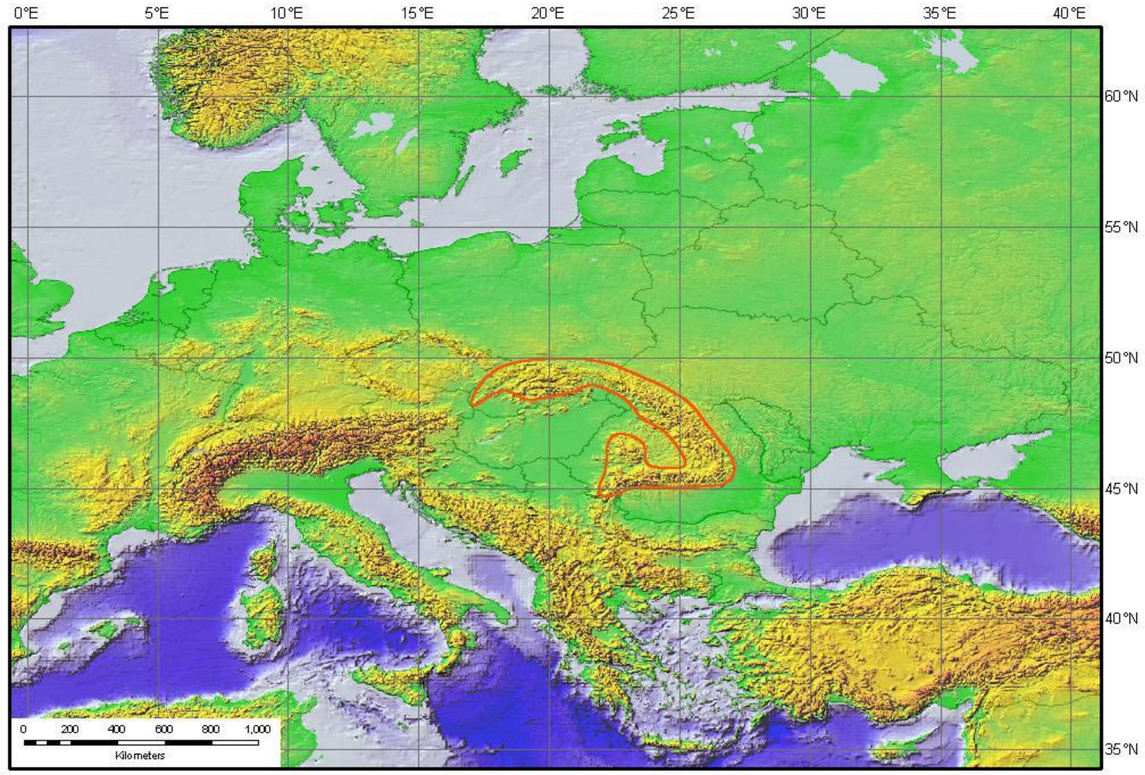

**FIGURE 1.** Location of the Carpathian Mountains in Europe.

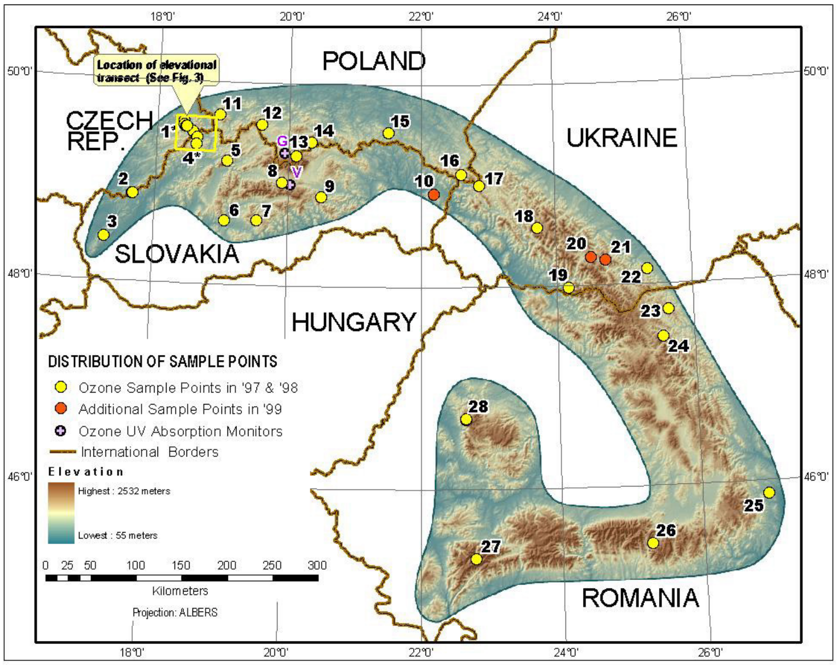

**FIGURE 2.** Location of monitoring sites in the Carpathian Mountains: 1*. Borova; 1*. Ostra; 1*. Luksiniec; 1*. Lysa Hora; 1*. Bili Kriz; 2. Javorina; 3. Male Karpaty; 4*. Riecky; 4*. Turkov; 5. Mala Fatra; 6. Kremnicke Vrchy; 7. Polana; 8. Vychodna; 9. Novoveska Huta; 10. Morske Oko; 11. Brenna; 12. Babia Gora; 13. Tatra; 14. Pieniny; 15. Magura; 16, Bieszczady; 17. Uzhoksky Pass; 18. Synevir ; 19. Kuzij; 20. Yablunitsa; 21. Kryvopilja; 22. Vizhnitsa; 23. Obcina Mare; 24. Rarau; 25. Magura O.; 26. Fundata; 27. Retezat; 28. Stana de Vale.

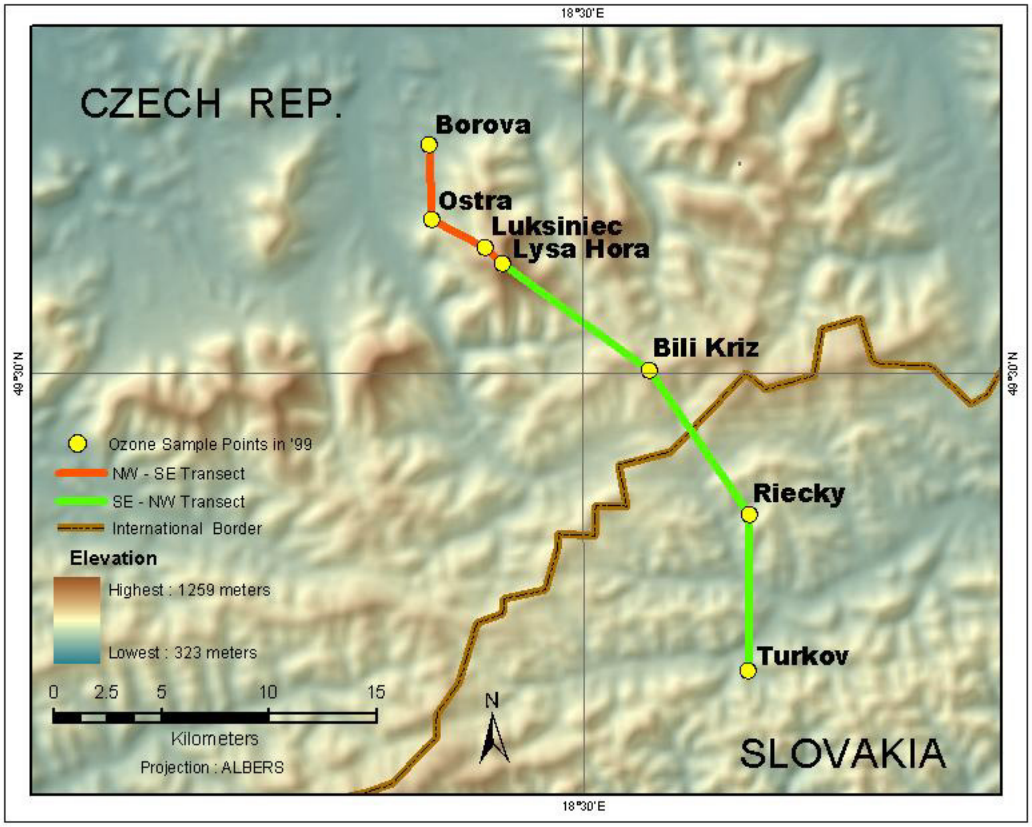

**FIGURE 3.** Elevational transects in the Morava-Silesian Beskid Mointains.

## Origin of DEM Data

The Digital Elevation Model (DEM) used for this study had a spatial resolution of 200 m, and was generated by mosaicing five individual DEMs, one part obtained from each Carpathian country. The final shape of the DEM is an effect of clipping of a much larger DEM data set to the outline of the Carpathian Mountains described in this study. The highest elevation point on the data was 2,532 m and the lowest point 55 m. All digital data sets were transformed to the same cartographic projection. An equal area projection of Albers had been selected with the central meridian of 22°E, and standard parallels of 45.5 and 48.5° N. These projection parameters ensured the most accurate measurements of area. The non-raster datasets including ozone measurement sites and weather stations as well as supporting data were converted into an ESRI Geodatabase format.

## Origin of Land Use Data

The digital land use data applied in this project was adapted from the CORINE Landcover Project of the European Union completed in 1998. The aim of the CORINE Program was to map the land use of Europe based on computer-assisted photo interpretation of satellite images by using ancillary data. The resulting land use data consists of 44 different land use types. The surface area of the smallest mapped unit is 25 ha (an area of 500 × 500 m). The interpretation results were scanned and integrated into a Geographical Information System (GIS) to create a map comparable in terms of its spatial accuracy with a topographic map of 1:100,000. For the purpose of this project focusing mostly on the main forest stands the land use classification was rasterized into 100 m resolution and reclassified into 2 categories – forest and no forest. The forest category consisted of the merge of all coniferous, deciduous and mixed forest initial classes.

## Testing of Geostatistical Analysts

A preliminary evaluation of Geostatistical Analyst was performed to decide which modeling deterministic methods (e.g., inverse distance weighting [IDW], Spline) or geostatistical methods (kriging, cokriging) along with their geostatistical algorithms (e.g., ordinary kriging, simple kriging, universal kriging, disjunctive kriging) and semivariogram models (e.g., spherical, exponential, Gaussian, J-Bessel) would provide the most accurate estimation of $O_3$ concentration surface. A total of 28 methods of data interpolation were applied and compared. The geostatistical interpolation algorithm that was selected to generate maps of ozone concentration distribution was based on statistical characteristics of each output surface based on comparison of cross-validation measures. Six cross-validation prediction error parameters were taken into account: mean, root-mean-square, root-mean-square standardized, average standard, mean standardized, and a difference between root-mean-square standardized and average standard errors for geostatistical algorithms. Only the first two statistical characteristics could be calculated for the IDW and spline methods.

Comparison of various parameters describing the quality of prediction did not provide a clear, univocal answer on what method most accurately estimated ozone spatial distribution. Based on the lowest prediction parameters error criteria, the best eight outputs were: spherical and K-Bessel models of cokriging with elevation as supplementary data set; best fitted representatives of spherical, exponential, Gaussian, and circular models of kriging; IDW with 15 neighbors; and spline with tension. Statistical prediction errors were similar for all final eight season average $O_3$ prediction candidate maps for the 1999 season. Due to a low-density sampling network, it was reasonable to adopt the most general (smooth) geostatistical approach. For that reason, as well as because of the generally very good rating of all the parameters of prediction error, the algorithm selected in spherical model of cokriging with the elevation as a secondary variable was recognized as the overall best and was utilized in this study to generate all maps of ozone predictions (Table 1).

A minimum of five nearest measurement points were used for calculating predicted values at any given location. Although the cross-correlation between ozone concentrations and elevation change did not appear strong and the monitoring network low sample site density, it provided a valid input for interpolation of $O_3$ results in some areas. A DEM for the Carpathian Mountains with 2 km resolution was applied for all semi-monthly measurement periods and annual average $O_3$ concentrations for the 1997 through 1999 for the summer seasons, and provided a second variable for the applied model. Interestingly, a DEM with a resolution of 200 m was available, but provided too much detail for models of ozone distribution. The DEM was resampled and several coarser resolutions were tested for their applicability as a cokriging covariant with the ozone data.

## Spatial and Temporal Distribution of Ozone Concentrations

The highest variability for annual $O_3$ average concentrations between the three years of study was detected in the vicinity of three Slovak sites (Vychodna, Polana and Novoveska Huta) and east of the Uzhoksky Pass in the Ukraine. Other Carpathian sites showed little variation in average $O_3$ concentrations between individual years (Fig. 4). The analysis of variance indicated significant differences between the sites, with the highest concentrations recorded for the Novoveska Huta and Polana sites (Slovakia), followed by Babia Gora (Poland). The lowest $O_3$ concentrations were recorded in Retezat (Romania) and Vychodna (Slovakia).

**TABLE 1**
**Evaluation of Various Modeling Approaches Tested within Geostatistical Analyst for the Carpathian Mountains**

| Geostatistical Interpolator | Model | Resolution of DEM (km) | Selected geostatistical input parameters[1] | | | | Parameters characterizing errors of estimation | | | | | |
|---|---|---|---|---|---|---|---|---|---|---|---|---|
| | | | Range (km) | Partial Sill | Nugget | Lag (km) | Mean | RMS | Avg. Std | Mean Std | RMS Std | RMS Avg. Std |
| *Kriging | Spherical | | 320 | 200 | 70 | 55 | -0.072 | 15.97 | 13.53 | -0.001 | 1.150 | 2.44 |
| Kriging | Spherical | | 200 | 80 | 132 | 60 | -0.001 | 16.42 | 13.87 | -0.001 | 1.144 | 2.55 |
| Kriging | Spherical | | 65 | 207 | 0 | 60 | -1.521 | 16.75 | 13.06 | -0.089 | 1.173 | 3.69 |
| Kriging | Spherical | | 69 | 205 | 0 | 35 | -1.235 | 17.32 | 12.98 | -0.067 | 1.208 | 4.34 |
| Kriging | Spherical | | 55 | 200 | 4.3 | 45 | -1.339 | 16.89 | 13.51 | -0.077 | 1.135 | 3.38 |
| *Kriging | Exponential | | 168 | 182 | 37 | 60 | -0.354 | 17.02 | 12.65 | -0.017 | 1.241 | 4.37 |
| Kriging | Exponential | | 60 | 196 | 10 | 45 | -1.120 | 17.02 | 13.81 | -0.067 | 1.137 | 3.21 |
| Kriging | Exponential | | 55 | 205 | 0 | 40 | -1.140 | 16.99 | 13.77 | -0.067 | 1.137 | 3.22 |
| Kriging | Gaussian | | 50 | 200 | 5 | 30 | -1.401 | 16.96 | 12.71 | -0.079 | 1.209 | 4.25 |
| Kriging | Gaussian | | 40 | 200 | 0.2 | 35 | -1.627 | 17.12 | 13.14 | -0.048 | 1.533 | 2.98 |
| Kriging | Gaussian | | 40 | 200 | 5 | 40 | -1.417 | 16.76 | 13.39 | -0.080 | 1.141 | 3.37 |
| *Kriging | Gaussian | | 30 | 200 | 10 | 45 | -0.339 | 16.36 | 14.47 | -0.021 | 1.107 | 1.89 |
| Kriging | K-Bessel | | 570 | 190 | 54 | 60 | -0.287 | 16.10 | 13.17 | -0.018 | 1.150 | 2.97 |
| Kriging | K-Bessel | | 55 | 208 | 10 | 45 | -1.307 | 17.07 | 13.04 | -0.073 | 1.176 | 4.03 |
| Kriging | J-Bessel | | 65 | 204 | 5 | 50 | -1.502 | 16.67 | 13.44 | -0.083 | 1.133 | 3.23 |
| Kriging | J-Bessel | | 75 | 202 | 5 | 45 | -1.517 | 16.98 | 13.05 | -0.082 | 1.190 | 3.93 |
| *Kriging | Circular | | 75 | 207 | 5 | 45 | -1.166 | 17.92 | 12.62 | -0.067 | 1.282 | 5.30 |
| Cokriging | Spherical | 20 | 65 | 207 | 5 | 35 | -1.496 | 16.79 | 13.09 | -0.091 | 1.165 | 3.70 |
| Cokriging | Spherical | 20 | 65 | 200 | 0 | 20 | -1.491 | 17.22 | 12.99 | -0.081 | 1.206 | 5.23 |
| **Cokriging | Spherical | 2 | 320 | 200 | 70 | 55 | -0.033 | 16.04 | 13.52 | -0.002 | 1.146 | 2.52 |
| Cokriging | Spherical | 2 | 75 | 202 | 5 | 35 | -1.419 | 17.58 | 12.89 | -0.083 | 1.233 | 4.69 |
| Cokriging | Gaussian | 2 | 75 | 215 | 5 | 35 | -1.280 | 18.15 | 11.92 | -0.073 | 1.439 | 6.23 |
| *Cokriging | K-Bessel | 2 | 75 | 193 | 10 | 35 | -1.280 | 16.91 | 13.06 | -0.100 | 1.178 | 3.85 |
| *IDW 15 | | | 220 | | | | -0.508 | 16.15 | | | | |
| IDW 10 | | | 120 | | | | -0.791 | 16.98 | | | | |
| IDW 12 | | | 95 | | | | -0.663 | 16.63 | | | | |
| *Spline | | | 220 | | | | -0.179 | 16.12 | | | | |

[1] Explanation of the selected geostatistical input parameters: Range - defines the diameter of neighborhood within which all measurements are related to each other; Sill - measures the value of spatial variation at the distance (range) where a maximum value is reached; Nugget - represents a sum of errors including: measurement errors, location error, uncertainty error, small scale irregularities; Lag Size - determines the distance up to which the correlation between sample points exists
* Selected best 8 candidates
**Final selection

*Note:* Cross-validation technique is frequently applied to assess the quality of geostatistical prediction. Among the parameters describing the error of estimation the Average Standard, Mean Standardized and RMS Standardized measures are used. The Average Standard error is simply the square root of the sum of all the kriging standard deviations divided by the total number of samples. Standardization of the Mean and RMS errors is based on cross-validation of the fitted variogram. In the process of normalization, the values of Mean and RMS errors are divided by the standard deviation of the estimated values, which can be also referenced as kriging standard error[11].

The model's ability to estimate digital GIS maps of $O_3$ distribution for the entire range of the Carpathian Mountains was limited because of the relatively small number of monitoring sites and the complex nature of air pollution distribution in mountainous terrain. Maps for individual semi-monthly periods over the 3 years of the study and the maps presenting annual averages for three years of the study (Fig. 5) show several consistent characteristics of $O_3$ distribution. Among these include a high spatial diversity of $O_3$ concentrations, especially in the Western Carpathians. In that area, where the network of monitoring sites is dense, neighboring sites had wide ranges of concentrations. Generally, similar spatial patterns of $O_3$ distribution occurred in the Carpathian Mountains during three years of the study.

Concentration of $O_3$ in the Carpathian Mountains may be dominated by multiple local sources of air pollution. No trend or anisotropy to its distribution was detected. The distribution of the highest and lowest $O_3$ concentration sites appeared to be geographically random. Whether it was really random, or whether there is an undetected pattern is uncertain. A denser network of sampling points is needed to determine the answer.

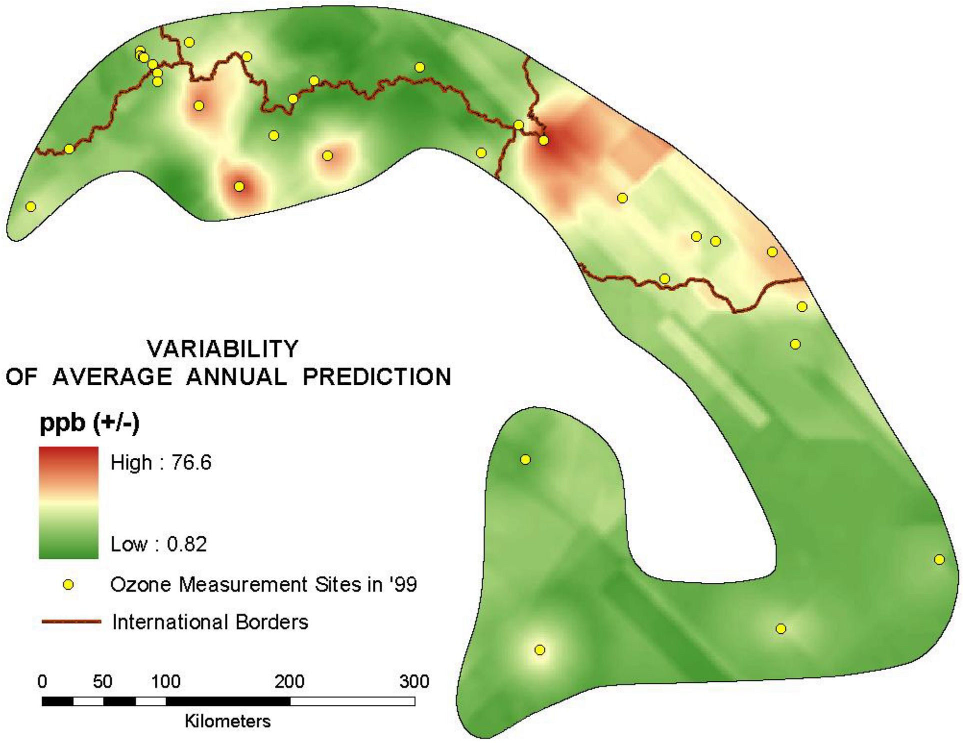

**FIGURE 4.** Year-to-year variability of $O_3$ concentrations on the Carpathian monitoring network.

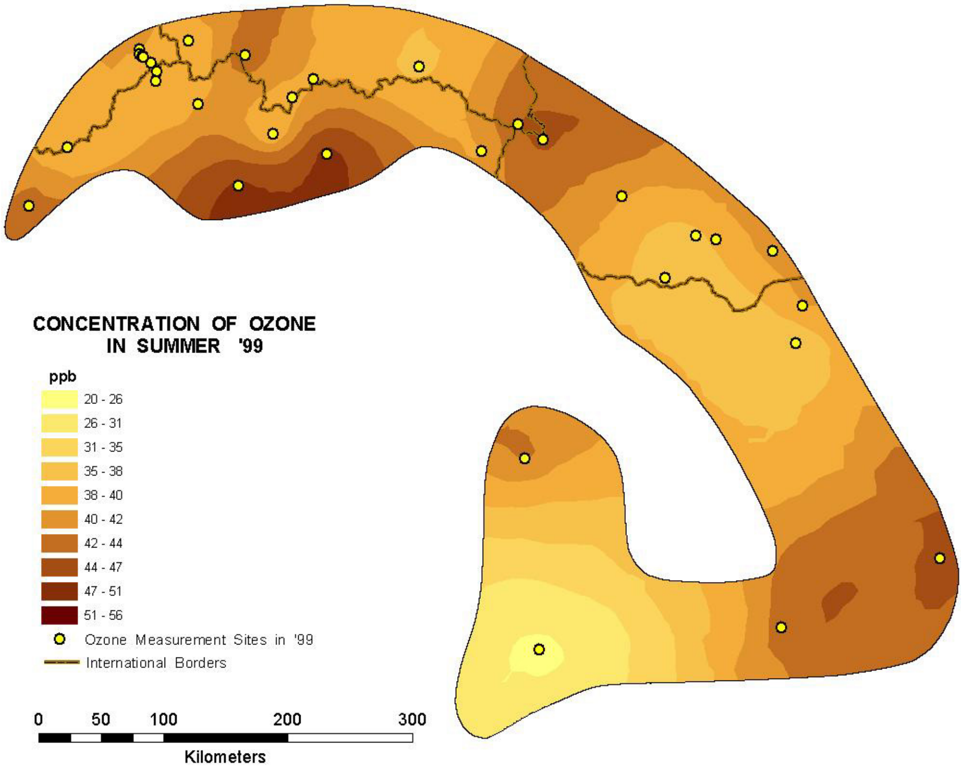

**FIGURE 5.** Model of spatial distribution of $O_3$ concentrations (ordinary cokriging with elevation as a co-variance) for 1999 season for the Carpathians.

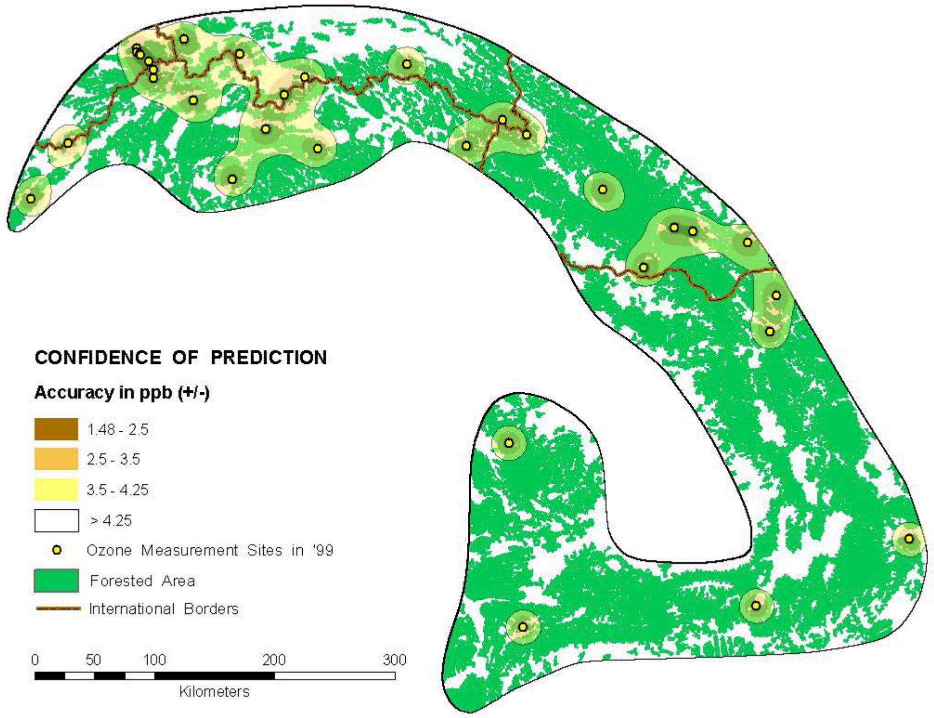

**FIGURE 6.** Confidence levels of the $O_3$ distribution predictions in the 1999 season in the Carpathian Mountains obtained with the Geostatistical Analyst.

## *Reliability of Ozone Concentration Estimates*

Since the number of monitoring sites was not sufficient to provide reliable results of estimation of $O_3$ concentration for the entire Carpathian Mountains area, an exercise was performed to indicate areas where additional $O_3$ measurement points should be located. The Geostatistical Analyst provides an algorithm that calculates standard error of a prediction. That allowed creating maps of prediction standard error of $O_3$ concentrations using the same interpolation algorithm and parameters that were used to generate the $O_3$ concentration maps. A range, or a threshold distance from monitoring sites up to which the estimated values were spatially autocorrelated to the measured values was set. That distance was used to delineate areas of estimated $O_3$ concentrations with sufficient confidence. The observed threshold was about 17 km. In other words, typically only within such a radius from an $O_3$ measurement site, a value of $O_3$ concentration could be confidently estimated. At such a distance from monitoring sites a prediction standard error was 4.25 ppb. Consequently, only the areas with a prediction standard error less than 4.25 ppb were considered to have satisfactory level of prediction confidence. The continuous reliability surface of the entire study area was then classified into four cartographical categories of potential error of prediction. The zones closest to the areas of the highest density of monitoring sites were considered satisfactory in terms of the density of the sites and consequently, the accuracy of prediction of $O_3$ concentrations. However, it has to be noted that even these areas have an inherited error of estimation $\geq \pm$ 1.5 ppb. This might be attributed to measurement errors and/or the effect of the microscale variation. Areas with a prediction standard error exceeding this observed threshold of 4.25 ppb covered about 80% of the entire study area. Forested areas outside of the prediction confidence limits constituted a large portion of the study area (Fig. 6). These are the target areas to locate the new monitoring sites in the future.

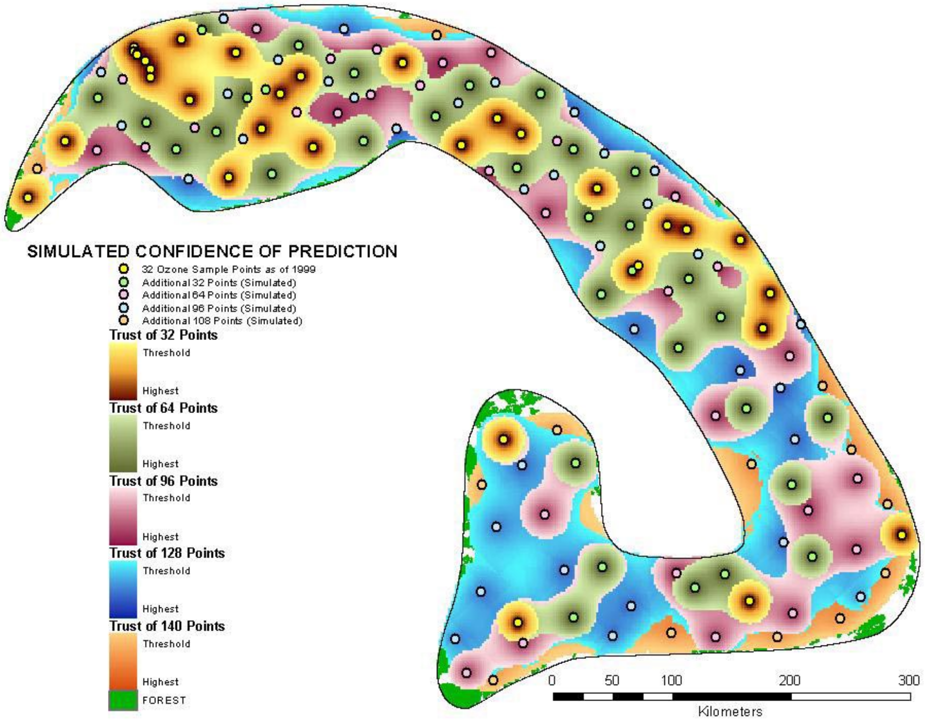

**FIGURE 7.** Approximate zones of reliability of $O_3$ predictions with the gradually increased density of monitoring sites in the Carpathians.

A simulation exercise was performed to determine the number of points needed to provide satisfactory predictions of ozone distribution for the entire area of the Carpathian Mountains (Fig. 7). The number of the original 32 sample points was doubled, tripled, and quadrupled. Locations of new sites were digitally placed in the forested areas characterized by the following features: (1) where the current study showed the highest level of $O_3$ concentration; (2) where variability of measurements was highest; and (3) where spatial gaps in the existing network of measurement points were present. The results indicated that as the number of monitoring sites for the Carpathian Mountains range increased to 140, confidence in predicting $O_3$ concentrations would cover about 99% of the entire forested area increased (Table 2). The efficiency of sampling for reliable estimates of ozone concentration reached an optimum with 64 sites, then gradually decreased with the increasing numbers of sites (Table 2). The decreasing efficiency beyond 64 sites was the result additional sites covering relatively small patches of unmonitored forests. Thus the most efficient sample design may not provide complete spatial coverage of the study area.

The network of measurement points could also be reduced if clear spatial trends or strong correlation with explanatory variables, e.g., elevation, are present. Strong relationships within the model reduce the number of points needed to separate spatial patterns from random or local variations in ozone concentrations.

## Evaluation of Monitoring Network and Performance of the Geostatistical Model

Surfaces of $O_3$ distribution for the Carpathian Mountains were calculated using passive ozone data and DEM (elevation used as a co-variant). Correlations between ozone and temperature were generally strongest, followed closely by a high negative correlation between ozone concentrations and relative humidity. High concentrations of ozone are likely to occur with high temperatures and low humidity[8,9]. It is possible that more reliable maps of the pollutant distribution could be generated if additional information on factors affecting ozone formation and transport were known.

**TABLE 2**
**Relationship between Number of Monitoring Sites in a Network and the Reliable Predicting Capability of Geostatistics for the Carpathian Mountains**

| Number of points in a network | Area of trustworthy prediction ($km^2$) | Average area of trustworthy prediction by each point ($km^2$) | % of the study area covered | % of the forested area covered | % of the forest covered by each sample point |
|---|---|---|---|---|---|
| 32 | 31,132 | 972 | 19.7 | 20.6 | 0.64 |
| 64 | 74,104 | 1158 | 46.9 | 51.0 | 0.80 |
| 96 | 108,356 | 1128 | 66.0 | 72.6 | 0.75 |
| 128 | 140,144 | 1094 | 88.7 | 92.0 | 0.72 |
| 140 | 152,203 | 1087 | 96.3 | 98.9 | 0.70 |

The number of monitoring sites established in the Carpathian Mountains was not adequate for developing reliable spatial estimates of $O_3$ concentrations over the entire mountain range. As the above simulation exercise (see Fig. 7 and Table 2) indicated, the number of monitoring sites for the Carpathian Mountains range would need to increase 4-fold before satisfactory confidence in predicting $O_3$ concentrations would be achieved for 92% of the entire forested area. Reliable estimates of the pollutant concentration distribution of all the forested areas in the Carpathian Mountains would require increasing the number of monitoring sites to about 140. In general a network of randomly distributed monitoring sites (1 site per 1,000 $km^2$) would provide reliable estimates of spatial distribution of ozone concentrations.

This sample density should not be automatically applied to other mountain ranges. In conditions of more predictable wind patters, such as during the summer conditions in the Californian Sierra Nevada Mountains, a less dense network could be adequate. In addition, if a clear correlation between elevation and $O_3$ concentrations was determined, the density of monitoring sites on a network could be significantly reduced. In general the more information regarding dynamics of air pollution transport in complex mountain terrain, such as transport patterns, re-circulation of air masses, etc., is available the fewer monitoring sites would be needed for the same reliability spatial estimates.

The simulated networks in our exercise were constructed without considering any particular sampling limitations such as proximity of roads, labor costs, safety of monitoring devices, etc. The final simulated network as well as the intermediate steps during the process of densification was not based on a regular grid for geometric optimization. Thus additional sample locations may be included for constructing an actual monitoring network.

## Sierra Nevada Mountains – Preliminary Analysis

### *Study Location and the Data Sources*

Sierra Nevada Mountains are located in California, the U.S. (Fig. 8), and are the most elevated mountain range in the 48 contiguous states. This mountain range contains extremely valuable natural and recreational resources. The Sierra Nevada contains only about half the land area as the Carpathians, but rise almost twice as high as mountains in the Carpathians. Consequently, the relief of the Sierra Nevada is much more dramatic and complex than that of the Carpathian Mountains.

Spatial surface estimation of the distribution of $O_3$ concentrations in the Sierra Nevada Mountains was conducted using the approach discussed for the Carpathians. Passive samplers were used to sample $O_3$ concentrations for 2-week periods between the beginning of May and the end of September in 1999. A major difference between the two studies was the much larger number of samplers used in the Sierra Nevada Mountains. Three times the number of air pollution

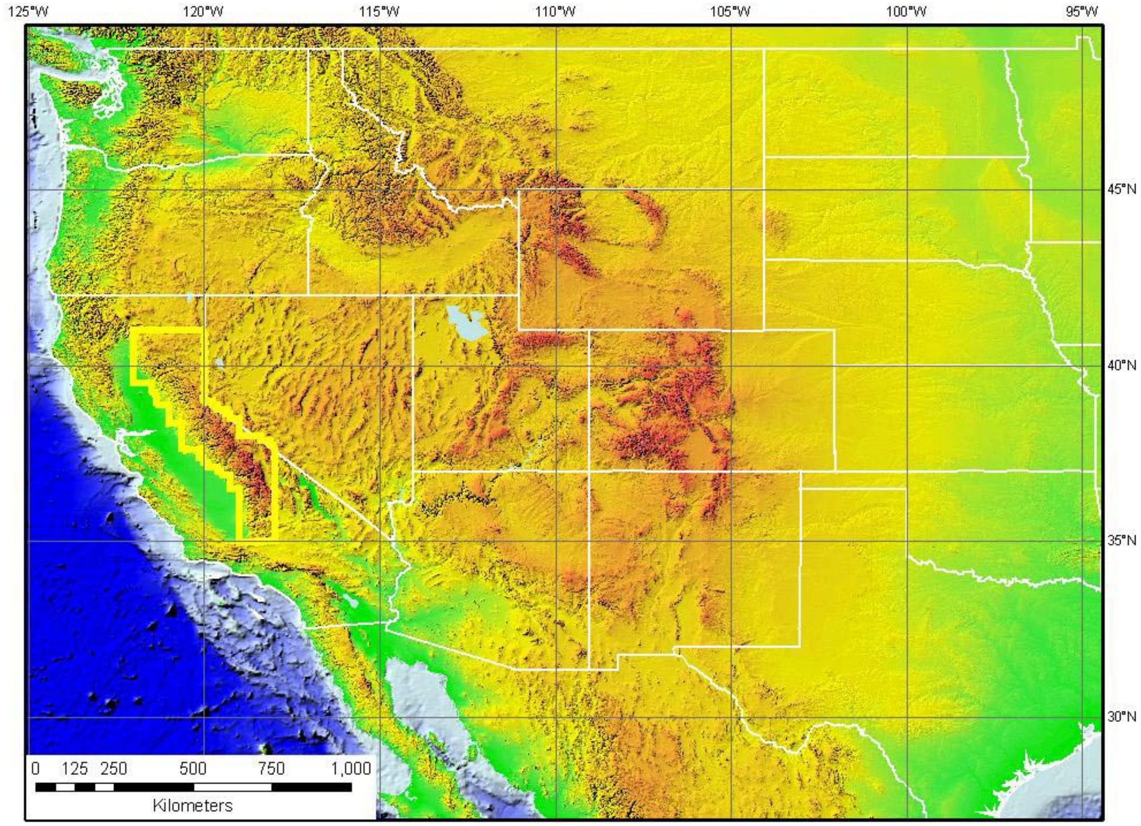

**FIGURE 8.** Location of the Sierra Nevada Mountains in the western U.S.

measurement sites (93) and weather data from 62 meteorological stations located in the study area were available for the analysis of the Sierra Nevada. The difference between the density of the monitoring networks for Carpathians and Sierra Nevada is illustrated on Fig. 9 which shows both study areas with their relevant $O_3$ measurement sites transformed to the same projection, scale and overimposed for the visual comparison.

All the air pollution measurement stations in the Sierra Nevada ranged in elevation from 223 to 2,796 m above sea level and were established and maintained by the USDA Forest Service. Meteorological data from 62 weather stations[10] provided information critical for this study. The meteorological monitoring stations also were located across a wide variety of elevations (52 to 2,551 m). These two data sets originated from two different sources and thus were independent of each other. This lack of spatial linkage did not reduce the ability of the Geostatistical Analyst to develop models describing $O_3$ concentration distribution. The majority of monitoring sites of both networks appear to be located near roads (Fig. 10), however all the measurement sites were situated at least 200 m away from a road to comply with the general principles of ambient air pollution sampling.

A DEM (elevation ranging from 19 to 4,415 m) was used as a collateral data to enhance the quality of the geostatistical estimation of the primary variable – $O_3$. The relevant, fine resolution elevation data for many topographic quadrangles was downloaded from the USGS web site http://edcwww.cr.usgs.gov/webglis/, resampled to a coarser resolution and merged into a single map. An effort was made to determine the optimal resolution of the DEM for this study. Depending on the purpose of the analysis, the capacity of a computer disk, and the speed of its processing unit, the resolutions from 30 to 2 km were found to be valuable for spatial surface estimation.

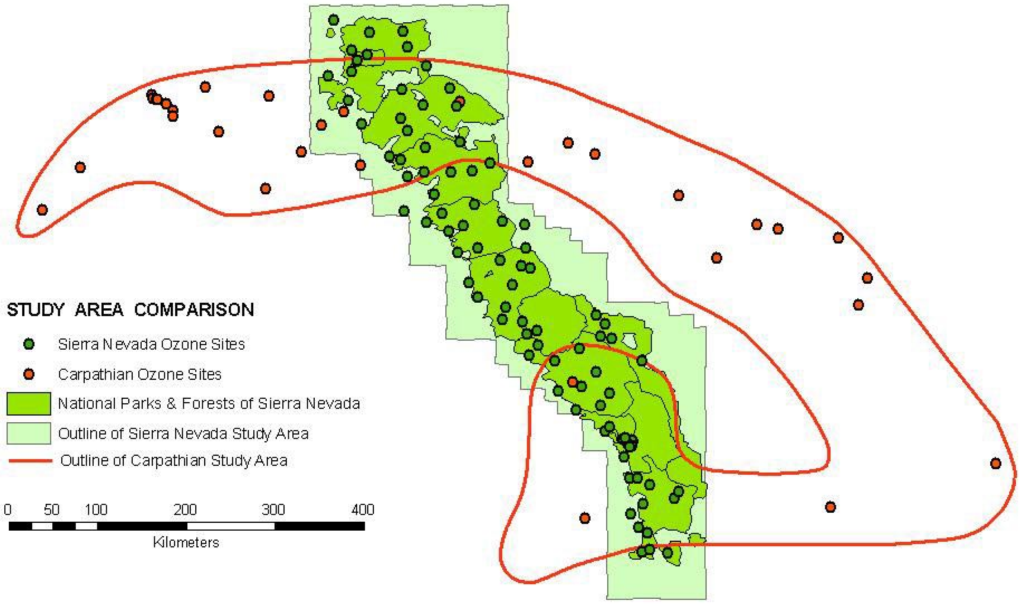

**FIGURE 9.** Comparison of sizes of the study areas and the density of measurement networks between the Carpathians and Sierra Nevada Mountains.

## Concentration of Ozone in the Sierra Nevada Mountains

There was a strong correlation between $O_3$ concentration and maximum temperatures in the Sierra Nevada. The Geostatistical Analyst also confirmed a strong correlation between temperature and elevation above sea level. Consequently, both the maximum temperature and the elevation were utilized as secondary and tertiary variables for the applied ordinary cokriging model. This discussion will be limited to the technical approach and preliminary results for the selected month of July 1999.

In order to assure that a dependable surface of maximum temperatures could be used for the model of the $O_3$ distribution, a temperature surface was generated using cokriging. The map of estimated maximum temperatures was based on the measurements from the weather stations cokrigged with the elevation data (DEM) applied as the secondary variable (Fig. 11). This map was very similar to the elevation map because of the strong obvious correlation between elevation and temperature that was taken into account by the cokriging method.

The model of $O_3$ concentrations for the month of July 1999 was estimated using the same approach. Data from the air pollution measurement sites was used as the primary variable while the previously created model of maximum temperatures was utilized as the secondary variable. Similarly to the $O_3$ study in the Carpathians, the ordinary cokriging method was applied to generate the output surface (Fig. 12) but additional information was used in the Sierra Nevada analysis that was not available for the Carpathian analysis. Thus, the resulting model of $O_3$ concentration distribution used all available $O_3$, temperature and elevation data.

Areas near Auburn and Placerville on the western side of the Sierras and around Mammoth Lakes and Bishop on the eastern side had the highest concentration of $O_3$ (exceeding 100 ppb) for the month of July. The lowest values of $O_3$ concentrations, around 30 ppb, were observed in the Lassen National Park and in the northern section of the Tahoe National Forest.

The estimated spatial surface of ozone concentrations (Fig. 12) is detailed and convincing for the study area, but reliability of prediction in the northeastern and southeastern parts of the map is questionable. This is due to a lack of measurement sites in these remote areas. Using similar methods as for the Carpathian ozone study (described above), the prediction standard

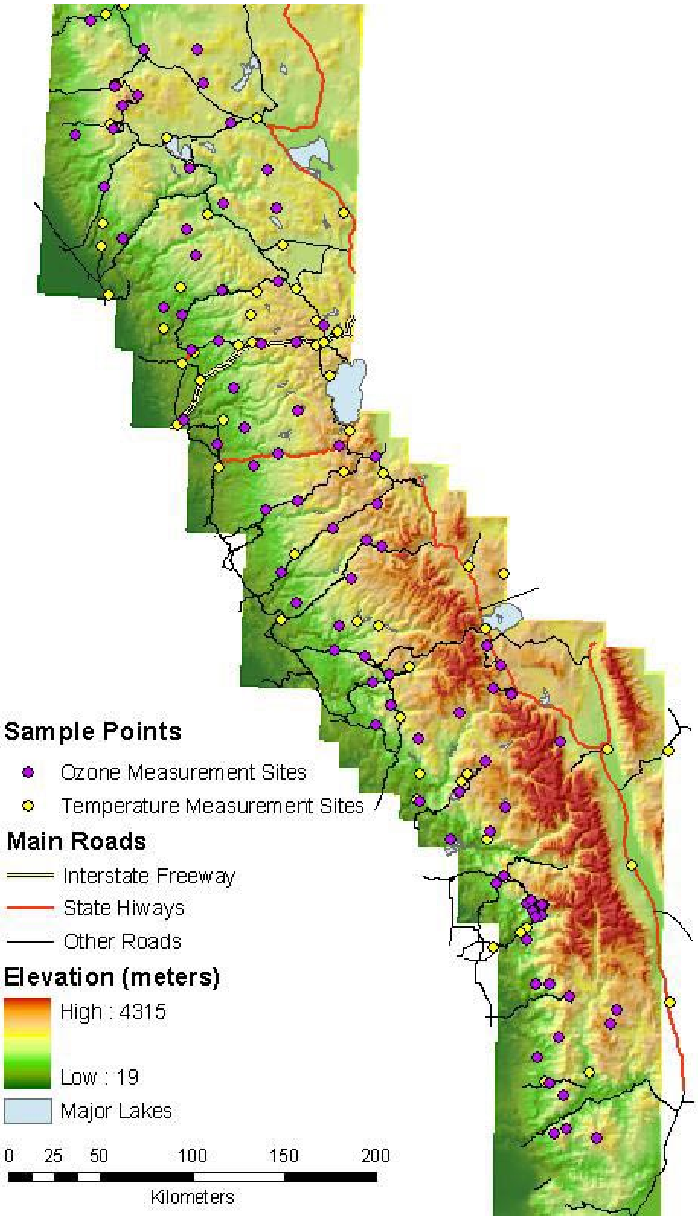

**FIGURE 10.** Networks of ozone and temperature measurement stations in Sierra Nevada Mountains.

error map was generated (Fig. 13).The lightest areas on the map represent the highest confidence of prediction of $O_3$ concentration. The dark areas represent areas with the low confidence. The estimated standard error of prediction was considered to be continuous over the entire study area.

Using the same methodology as described for the Carpathians and based upon the semivariogram analysis, it was found that those $O_3$ concentrations could be confidently estimated within a radius of about 19 km from the nearest measurement site toward the direction where the extrapolation techniques would be required. The value of prediction standard error at that distance was about ±12 ppb. This value was used to establish the final area of confident prediction. A rough outline of this area shows that most of the study area of the Sierra Nevada is adequately covered (Fig. 14).

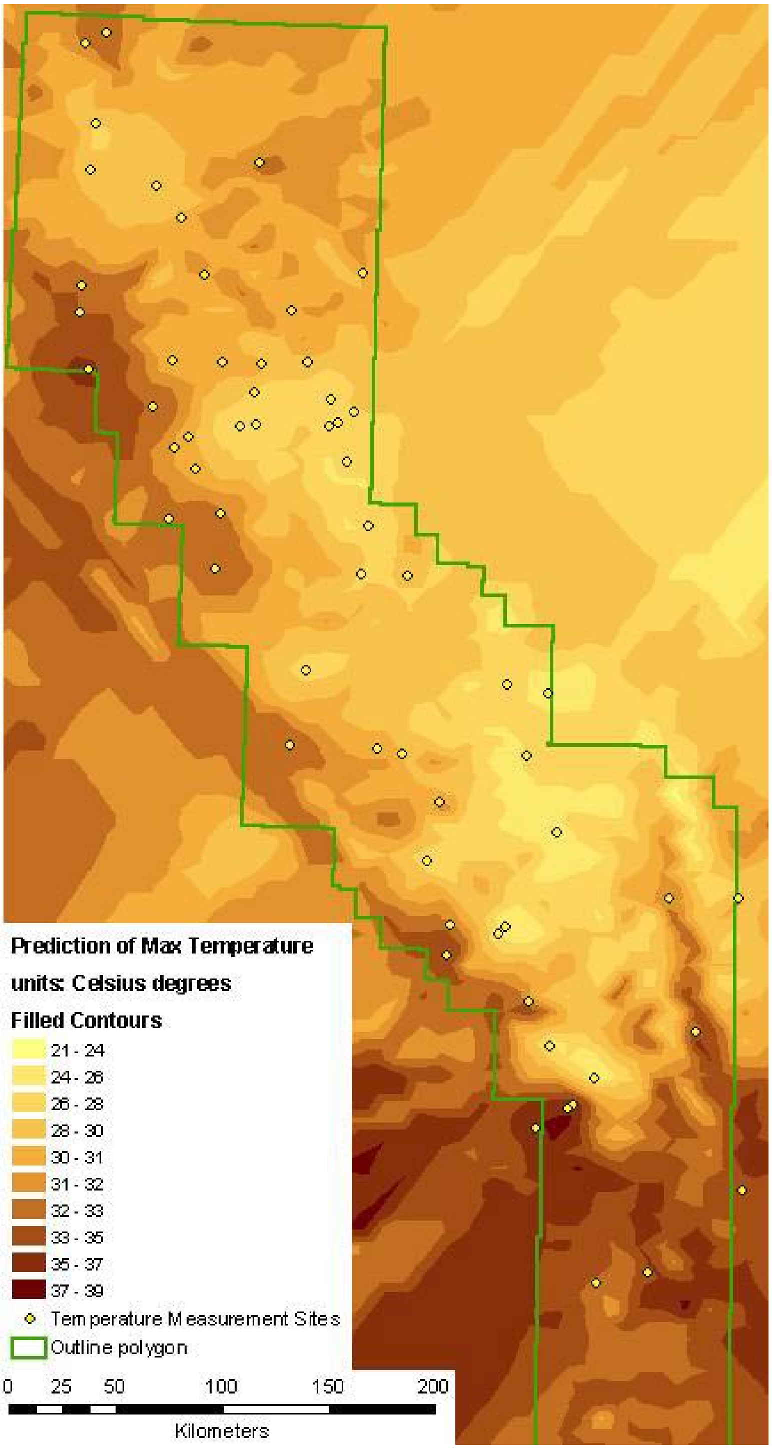

**FIGURE 11.** Maximum temperatures in July 1999 based on cokriging with elevation as the secondary variable.

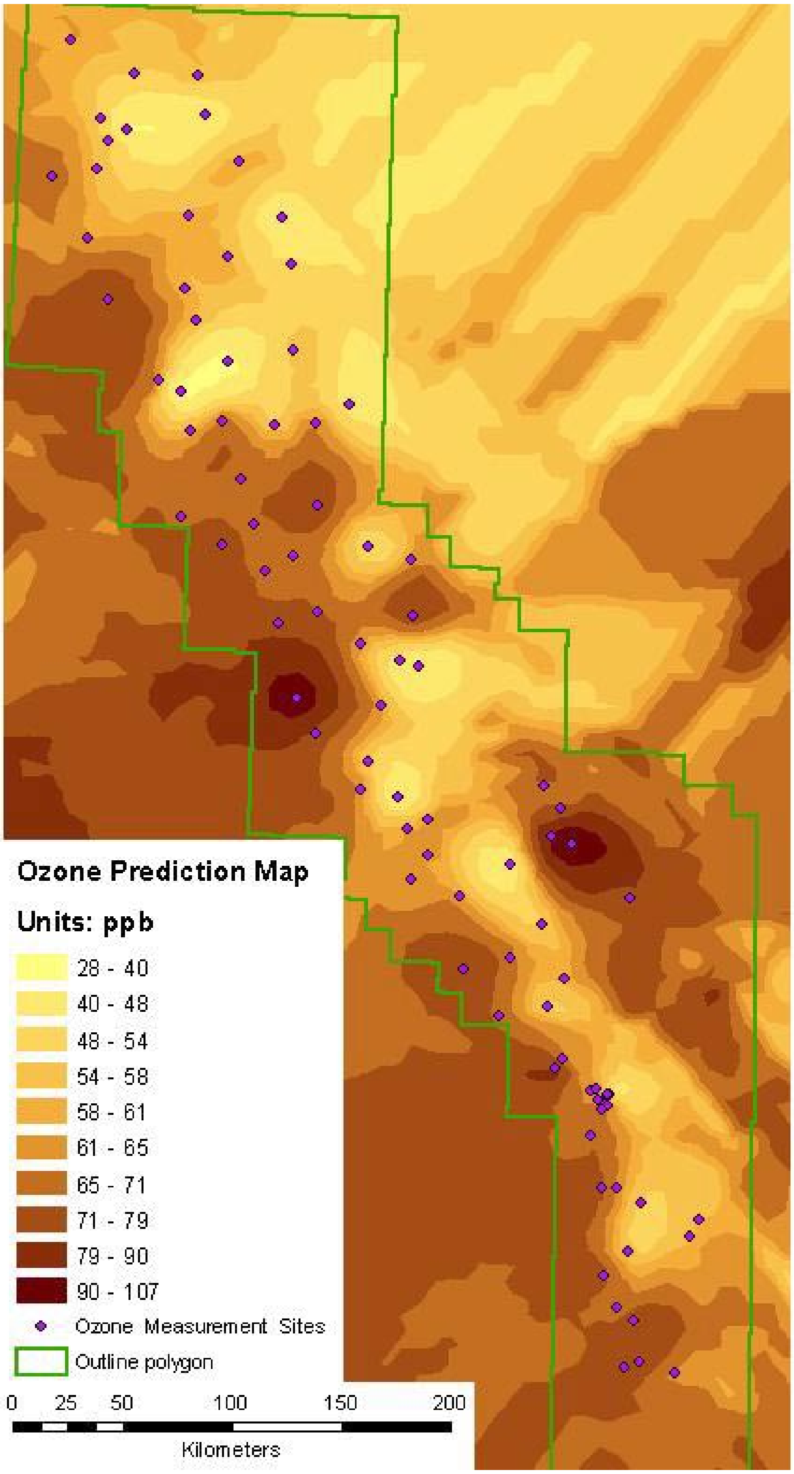

**FIGURE 12.** Prediction of ozone concentration based on cokriging of ozone, maximum temperature and elevation.

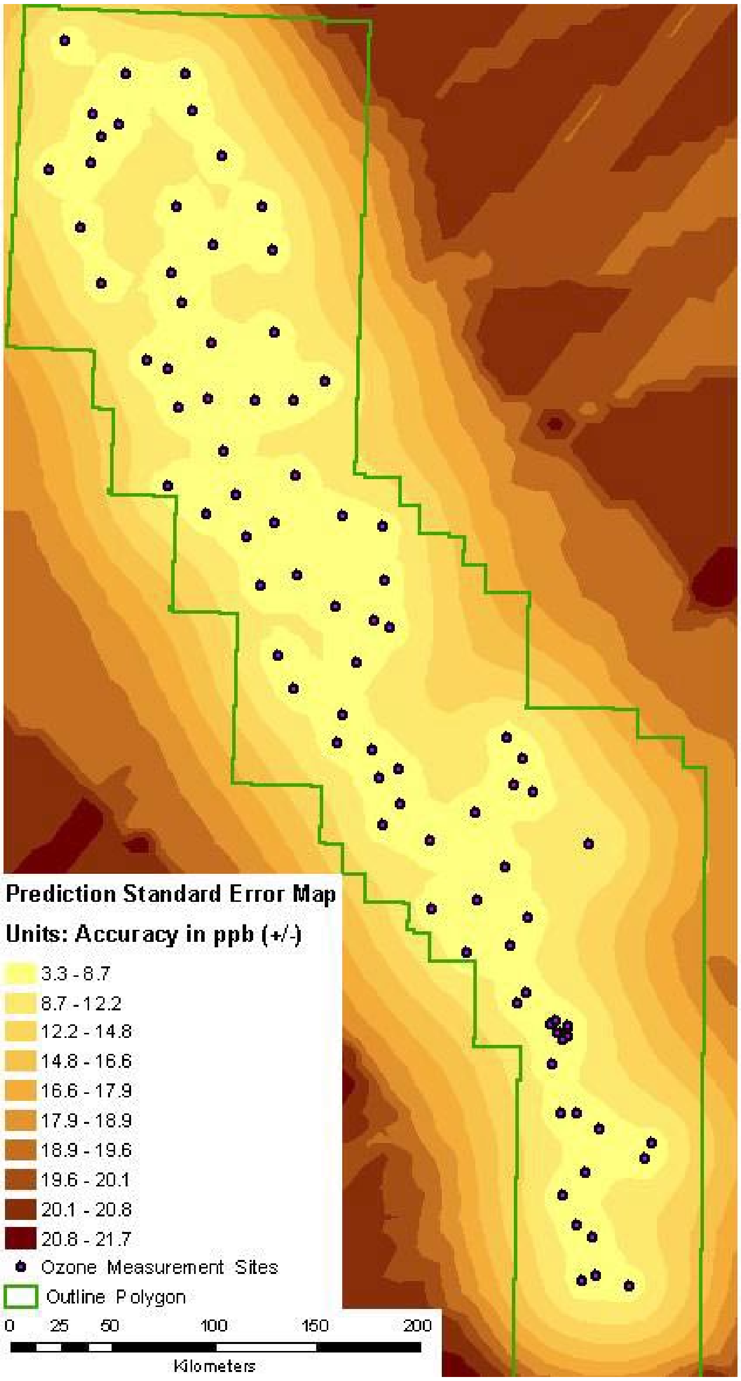

**FIGURE 13.** Potential standard error of estimation of ozone concentration in Sierra Nevada Mountains.

## CONCLUSIONS

The results of these parallel studies suggest that the density of the measurement sites network in the Carpathians was inadequate. Conversely almost 94% of the study area was reliably estimated for the Sierra Nevada, although the distribution of the measurement sites could still be improved (there was clearly insufficient number of sites on the eastern side of the mountain range). The esti-

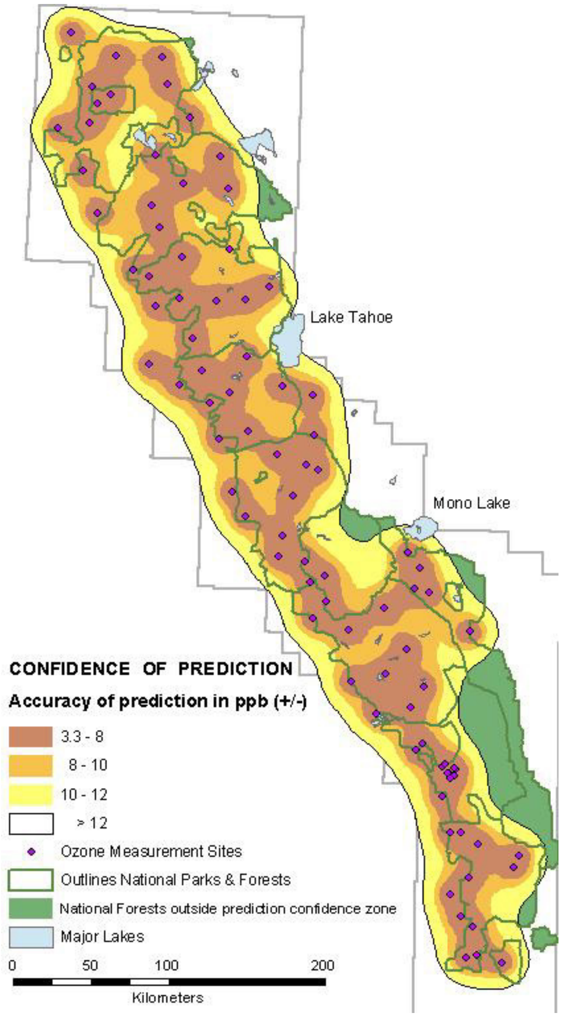

**FIGURE 14.** Approximate zone of reliable prediction of ozone concentration in July 1999 overimposed on National Parks and Forests in Sierra Nevada Mountains.

mated area of the reliable prediction covered by each sample point in the Sierra Nevada was on average 488 km$^2$, while the actual average area covered by each sampling point was 514 km$^2$, a very close match. Similar estimates for the Carpathian study were 973 km$^2$ and 4,937 km$^2$, indicating a great disparity between the sample size needed and that obtained. Increasing the number of monitoring sites in the Carpathian study to 140 would change the relevant values to 1087 and 1128 km$^2$. The availability of the weather data and presence of strong correlations between maximum temperatures and elevation with $O_3$ contributed to much lower sampling site densities being needed to obtain reliable spatial surface estimates in the Sierra Nevada compared with the Carpathian Mountains.

## ACKNOWLEDGEMENTS

We would like to thank the Environmental Systems Research Institute for allowing us to use the Geostatistical Analyst and for technical help provided by their software engineers. We also thank field staff in the Carpathian Mountains and the Sierra Nevada for timely and careful collection of passive samplers. The USDA Foreign Agricultural Services, the USDA Forest Service and the California Air Resources Board provided financial support for these studies.

## References

1. Zeigler, M. (1999) *Modeling Our World. The ESRI Guide to Geodatabase Design*. ESRI Press, Redlands, CA.
2. Lang, L. (1998) *Managing Natural Resources with GIS*. ESRI, Redlands, CA.
3. Johnston, K., Ver Hoef J., Krivoruchko K., and Lucas, N. (2001) *Using ArcGIS Geostatistical Analyst*. ESRI, Redlands, CA.
4. Phillips, D.L., Lee, E.H., and Herstrom, A.A. (1997) Use of auxillary data for spatial interpolation of ozone exposure in southeastern forests. *Environmetrics* **8**, 43–61.
5. Simpson, D. (1991) *Long Period Modeling of Photochemical Oxidants in Europe*. EMEP, Cooperative Programme for Monitoring and Evaluation of the Long Range Transmission of Air Pollutants in Europe, Meteorological Synthetizing Centre – West, The Norwegian Meteorological Institute, Oslo, Norway 44 pp.
6. Koutrakis, P., Wolfson, J.M., Bunyaviroch, A., Froelich, S.E., Hirano, K., and Mulik, J.D. (1993) Measurement of ambient ozone using a nitrite-saturated filter. *Anal. Chem*. **65**, 210–214.
7. Bytnerowicz, A., Godzik, B., Frączek, W., Grodzińska, K., Krywult, M., Badea, O., Barancok, P., Blum, O., Černy, M., Godzik, S., Mankovska, B., Manning, W. J., Moravčik, P., Musselman, R. M., Oszlanyi, J., Postelnicu, D., Szdżuj, J., Varšavova, M., and Zota, M. Distribution of ozone and other air pollutants in forests of the Carpathian Mountains in central Europe. *Environ. Pollut*. (in press).
8. Van Ooy, D.J. and Carroll, J.J. (1995) The spatial variation of ozone climatology on the western slope of the Sierra Nevada. *Atmos. Environ*., **29**, 1319–1330.
9. Bolhar-Nordenkampf, H. (1999) Ecophysiological analysis to evaluate the tree-specific ozone risk map of Austria with regard to the local patterns of stress factors. Institut fur Okologie und Naturschutz, Universitat Wien, Austria.
10. NOAA (1999) National Climatic Data Center, Daily Surface Data, October 8, 1999. Ashville, North Carolina.
11. Cressie, N. (1993) *Statistics for Spatial Data*. John Wiley & Sons, Inc. New York.

### BIOSKETCH

**Witold Fraczek** has MS in hydrology from the University of Warsaw, Poland (1976) and another MS in environmental monitoring/remote sensing from the University of Wisconsin, Madison (1988). Since late 1988 he is working at Environmental Systems Research Institute (ESRI) in Redlands, CA.

**Andrzej Bytnerowicz** received his Ph.D. in Natural Sciences in 1981 from the Silesian University in Katowice, Poland. He is an Ecologist at the Pacific Southwest Research Station, USDA Forest Service, Riverside, California and an Adjunct Professor at the Department of Environmental Sciences of the University of California in Riverside. He is a member of editorial boards of “Environmental Pollution” and “Terrestrial Environmental Toxicology” of TheScientificWorld. His main research interests are distribution and deposition of air pollutants in mountain forest ecosystems and effects of ozone and nitrogenous air pollutants on plants. Since 1991 Dr. Bytnerowicz has coordinated and actively participated in several projects on effects of air pollution and climate change in forests of California and Central Europe. He is an author of more than 100 scientific papers and book chapters.

**Michael Arbaugh** holds a BS in biology and an MS in statistics from the University of California, Riverside and a Ph.D. in Forest Sciences from Colorado State University (1995). He is currently working at the USDA-Forest Service Riverside Fire Laboratory in California, on air pollution and fuels management tradeoffs effects on western forests.
